# Long-term clinical and biomarker observations following rituximab exposure in two siblings with *CSF1R*-related leukoencephalopathy: a preliminary familial case series

**DOI:** 10.1038/s43856-026-01936-5

**Published:** 2026-09-30

**Authors:** Ramojus Balevicius, Lidija Marija Smertinaite, Mourad Kourie, Zbigniew Wszolek, Goda-Camile Mickeviciute, Robert Harris, Tobias Granberg, Nadezda Zhuravleva, Jinming Han, Virginija Danylaite Karrenbauer

**Affiliations:** 1 Department of Clinical Neuroscience, Karolinska Institutet, Stockholm, Sweden; 2 Department of Biological Models, Institute of Biochemistry, Life Sciences Center, Vilnius, Lithuania; 3 Vrinnevis Hospital, Norrköping, Sweden; 4 Department of Neurology, Mayo Clinic, Jacksonville, FL USA; 5 Versicherungsanstalt für Eisenbahnen und Bergbau, Gesundheitseinrichtung, Bad Hofgastein, Austria; 6 Center for Molecular Medicine, Karolinska University Hospital, Stockholm, Sweden; 7 Department of Neuroradiology, Karolinska University Hospital, Stockholm, Sweden; 8 CENTOGENE GmbH, Rostock, Germany; 9 Department of Neurology, Xuanwu Hospital, Capital Medical University, Beijing, China; 10 Department of Neurology, Karolinska University Hospital, Stockholm, Sweden

**Keywords:** Neurodegeneration, Disease genetics, Demyelinating diseases

## Abstract

**Background:**

Colony-stimulating factor 1 receptor (*CSF1R*)-related leukoencephalopathy (CRL) is a rare microgliopathy, characterized by progressive neurodegeneration. Impaired *STAT3* signaling reduces microglial numbers and limits their ability to clear cellular debris, leading to persistent inflammation and brain tissue damage that activates peripheral CD20⁺ B cells. These cells migrate into the CNS and secrete interleukin-6 (IL-6), amplifying inflammation and further disrupting STAT3 signaling in a self-reinforcing cycle that drives white-matter injury.

**Methods:**

We describe two siblings from a single family, both carrying the same heterozygous *CSF1R* variant (c.2562 T > A, p.Asn854Lys), who received four infusions of rituximab (RTX;500 mg) on a compassionate, off-label basis between 2014-2017. The long-term disease trajectory was evaluated using standardized clinical rating scales and brain magnetic resonance imaging (MRI). As a secondary biomarker outcome, serum and CSF neurofilament light chain (NfL) concentrations were assessed.

**Results:**

In both siblings, RTX exposure was followed by EDSS stability at 6.5 and 7.0, respectively, over 5 years, with mixed trajectories in other functional and cognitive domains. Over the 23-year MRI follow-up period, patients developed cerebral atrophy. During the post-treatment period, no new focal lesions were observed on brain MRI, and levels of CSF-NfL and CSF-CXCL13 were normalized.

**Conclusions:**

In this preliminary familial case series of two siblings carrying the same *CSF1R* variant, RTX exposure was followed by long-term clinical stability and favorable biomarker trajectories. Patient 1 had a pre-treatment disease course of approximately 13 years, indicating an inherently slow disease progression that cannot be entirely separated from the treatment effect in an uncontrolled observation.

**Figure:**
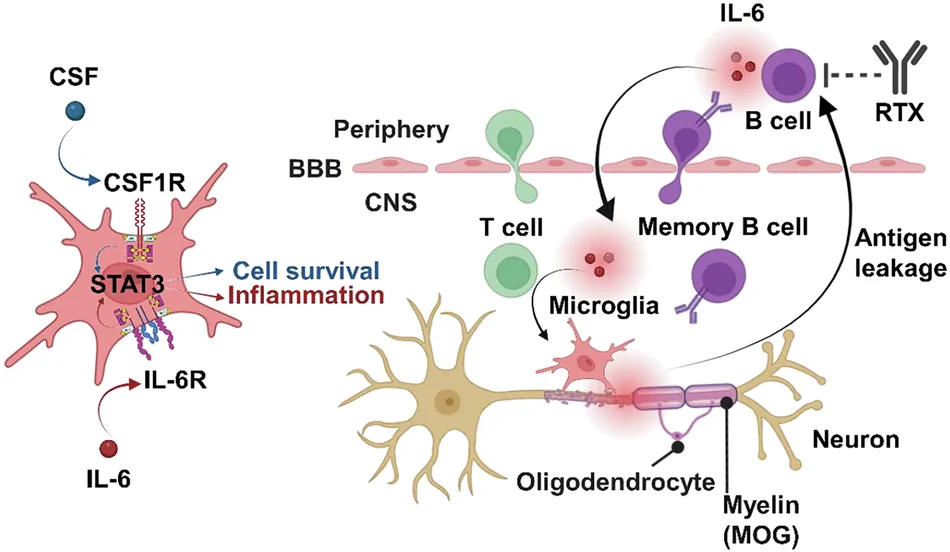

## Plain Language Summary

CSF1R-related leukoencephalopathy (CRL) is a rare inherited brain disorder that gradually affects movement, thinking, and behavior by damaging brain tissue. Research suggests that certain immune cells may contribute to inflammation in the brain. We report the experiences of two sisters with CRL who carried the same disease-causing change in the CSF1R gene. They received four infusions of rituximab, a treatment that reduces the number of these immune cells, and were followed for several years with neurological assessments, brain scans, and blood tests. After treatment, their symptoms remained stable or improved, no new areas of brain damage were seen on scans, and blood test results improved. Larger studies involving more people with CRL are needed to determine whether this treatment could benefit others with the condition.

## Introduction

Colony-stimulating factor 1 receptor (*CSF1R*)-related leukoencephalopathy (CRL) is a rare, progressive neurodegenerative disorder characterized by diffuse white matter degeneration, cognitive impairment, motor dysfunction, and behavioral changes^1^. CRL represents a spectrum of microgliopathies in which the central pathological driver is microglial dysfunction, the resident immune cells of the central nervous system (CNS). Microglia play a critical role in maintaining neural homeostasis, including the clearance of apoptotic cells, regulation of synaptic pruning, and support of oligodendrocyte survival and myelination. In CRL, heterozygous or homozygous mutations in the *CSF1R* gene compromise these functions, leading to widespread neuroinflammation and progressive white matter injury^2^.

The *CSF1R* gene encodes a transmembrane tyrosine kinase receptor (CSF1R) essential for the development, survival, and function of microglia and peripheral mononuclear phagocytes^1^. Activation of CSF1R occurs upon binding to its ligands – colony-stimulating factor 1 (CSF1) or interleukin-34 (IL-34) – inducing receptor dimerization and autophosphorylation^3^. These phosphorylated tyrosine residues serve as docking sites for downstream effectors, including PI3K and STAT3^4^. Upon phosphorylation, STAT3 dimerizes and translocates to the nucleus, initiating the transcription of genes that regulate cell survival, proliferation, and immune homeostasis^5^. Consequently, impaired STAT3 signaling precipitates microglial dysfunction, reduces microglial density, and leads to compromised white matter integrity. In CRL, these proinflammatory microglial phenotypes exacerbate neuroinflammation, contributing to demyelination, axonal injury, and progressive neurodegeneration. Furthermore, *CSF1R* dysfunction extends to peripheral macrophages and monocytes, resulting in systemic immune dysregulation^6^.

The *CSF1R* mutation’s effects on microglial survival are further compounded by the resulting inflammatory response. Compromised microglial survival and the resulting reduction in microglial density lead to the accumulation of cellular debris, impaired phagocytosis, and reduced clearance of apoptotic cells, which trigger a secondary inflammatory response. Peripheral B cells are then activated and infiltrate the CNS, where they perpetuate chronic inflammation through the secretion of interleukin-6 (IL-6)^7^. IL-6 acts as a potent signaling molecule that modulates both innate and adaptive immunity, often exhibiting a strong proinflammatory profile^8^. Specifically, IL-6 promotes neuroinflammation and neurodegeneration via the canonical STAT3 signaling pathway. While STAT3 is essential for oligodendrocyte regeneration and remyelination^9^, its chronic dysregulation in an inflammatory context is deleterious. In CRL, this IL-6–mediated inflammatory state may further destabilize STAT3 signaling, compounding the effects of *CSF1R* haploinsufficiency and exacerbating white matter injury and demyelination.

Rituximab, a monoclonal antibody targeting CD20, mediates the depletion of CD20⁺ B cells. A specific subset of these cells, including activated and memory B cells, is a potent source of IL-6, particularly in autoimmune or inflammatory states^10^. We hypothesized that depletion of IL-6–producing B-cell subsets may attenuate this inflammatory cycle and theoretically support more homeostatic STAT3 signaling, with potential downstream effects on oligodendrocyte and microglia survival. Direct evidence for this mechanism in CRL is currently lacking, and the present report was intended to motivate further mechanistic and clinical investigation into this pathway. In the context of CRL, targeting B-cell-mediated IL-6 production represents a complementary therapeutic strategy to mitigate neuroinflammation and support white matter repair. Furthermore, the crosstalk between central and peripheral immune compartments may yield novel insights into disease-modifying therapies.

To explore this hypothesis in a clinical context, we describe long-term clinical, radiological, and biomarker observations in two siblings with genetically confirmed CRL who receive RTX on a compassionate, off-label basis. We find stabilization of disability progression, absence of new focal MRI lesions, and favorable biomarker trajectories following RTX exposure, supporting further investigation of B-cell-directed therapeutic strategies in CRL. This work is presented as a preliminary, hypothesis-generating familial case series in an effort to draw attention to the therapeutic potential of anti-CD20 strategies in CRL treatment.

## Methods

### Patients

#### Inclusion and exclusion criteria

Inclusion criteria: CRL diagnosis according to Konno et al.^11^; attending Karolinska University Hospital, Neurology clinic; patients have received RTX treatment; patients have written informed consent. Exclusion criteria: withdrawal of informed consent; not fulfilling CRL diagnostic criteria.

#### Recruited patients

Two adult female siblings of Finnish Kale descent, both carrying the same heterozygous *CSF1R* missense variant (c.2562 T > A, p.Asn854Lys), were initially diagnosed with multiple sclerosis (MS)^12^. Following identification of the *CSF1R* variant, the diagnosis was revised to adult-onset leukoencephalopathy with axonal spheroids and pigmented glia (ALSP/CRL).

### Outcomes, exposure, bias

Due to long-term follow-up and differential data composition prior to treatment and post-treatment study periods, we cannot deliver a formal pre-/post-treatment comparison; the data should be interpreted as a descriptive long-term post-exposure trajectory.

#### Primary outcome

Evaluation of disease progression using clinical scales and magnetic resonance imaging (MRI). The baseline clinical data presented for the pre-treatment period are derived from contemporary routine clinical records. Standardized assessments using the rating scales listed below were only initiated from 2020 onwards, that is, 3–6 years after the final RTX infusion in each patient. Because no validated, disease-specific rating instrument exists for CRL, we assembled a multimodal assessment battery designed to capture the heterogeneous clinical features of the disorder: parkinsonism and gait disturbance, motor neuron involvement, neuropsychiatric symptoms, global disability, cognition, and patient-reported quality of life. Parkinsonian features were evaluated using the Movement Disorder Society–sponsored revision of the Unified Parkinson’s Disease Rating Scale (MDS-UPDRS)^13^, which comprises four parts: Part I (non-motor experiences of daily living), Part II (motor experiences of daily living), Part III (motor examination), and Part IV (motor complications). The modified Hoehn and Yahr stage^14^ and the Schwab and England Activities of Daily Living scale were applied alongside the MDS-UPDRS as freestanding measures. The Amyotrophic Lateral Sclerosis Functional Rating Scale (ALSFRS-R)^15^ was included to assess upper motor neuron–type weakness and bulbar function. The Hospital Anxiety and Depression Scale (HADS)^16^ was used to monitor neuropsychiatric burden, and the Expanded Disability Status Scale (EDSS)^17^ was retained because both patients had originally been assigned an MS diagnosis and longitudinal EDSS data were available from routine care, providing the only standardized measure with comparable pre-treatment values. Cognitive status was evaluated with the Mini-Mental State Examination (MMSE)^18^ and the Symbol Digit Modalities Test (SDMT)^19^. Quality of life was estimated with the MS Impact Scale (MSIS-29)^20^ and EuroQol-5D (EQ-5D / VAS)^21^.

**Fig. 1:**
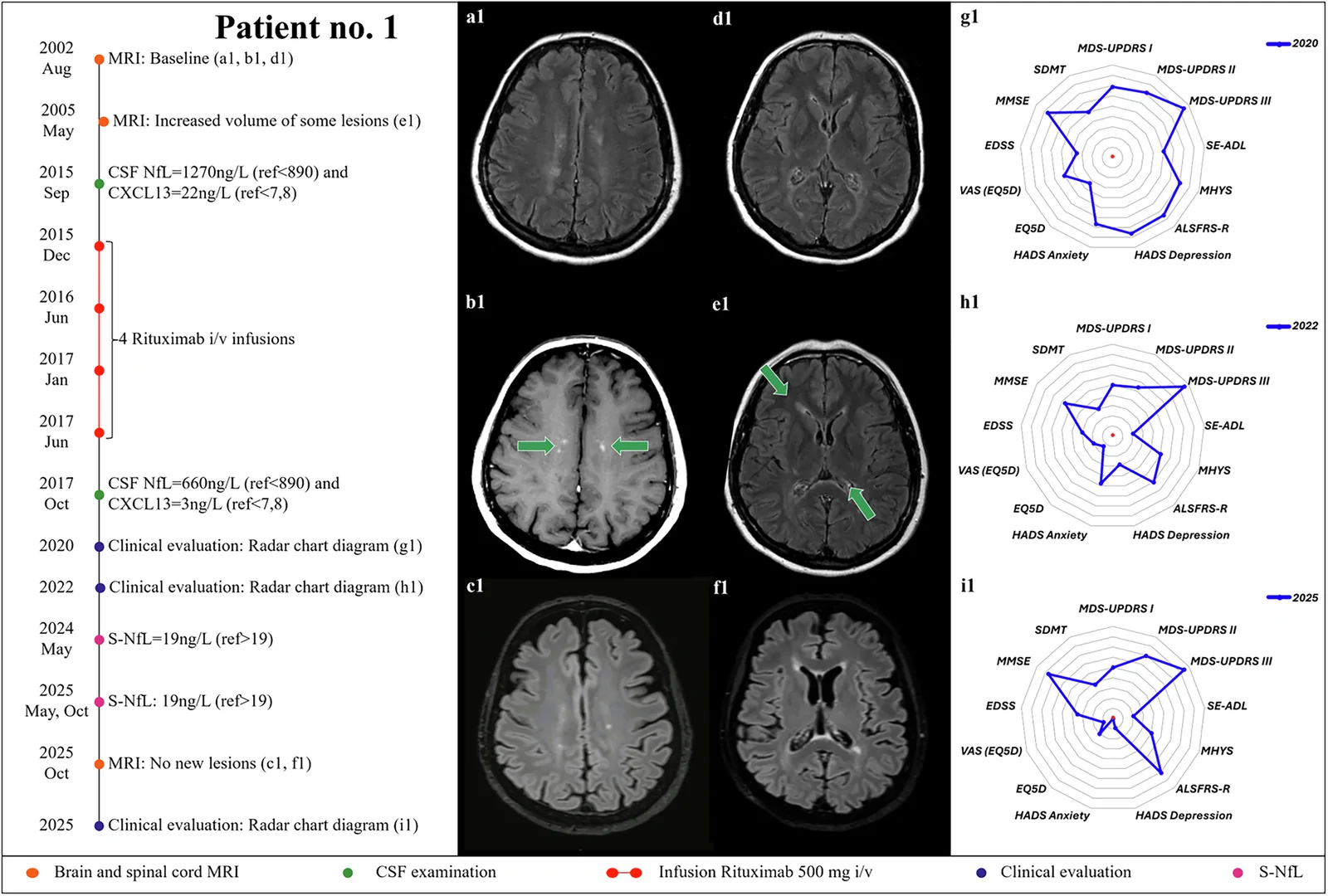
Disease evolution and treatment timelines for patient 1. **a1**, **d1** First brain MRI, FLAIR with multiple periventricular lesions in 2002; **b1** T1-weighting imaging showing contrast-enhancement in several lesions (green arrows, 2002); **e1** Two new periventricular lesions (non-enhancing, green arrows) at 3-year follow-up (2005); **c1**, **f1** 23-year MRI follow-up with no additional lesions but with widening of cerebrospinal spaces due to atrophy. Radar chart diagrams with data of multimodal clinical evaluations performed in years **g1** 2020; **h1**2022; **i1** 2025. Abbreviations: ALSFRS ALS Functional Rating Scale, CXCL Chemokine (C-X-C motif) ligand, EDSS Expanded Disability Status Scale, EQ5D EuroQol-5D standardized instrument for measuring generic health status, HADS Hospital Anxiety Depression Rating Scal, Ig immunoglobulin, IL interleukin, L liter, MDS-UPDRS Movement Disorder Society–Unified Parkinson’s Disease Rating Scale, mg milligram, MHYS Modified Hoehn and Yahr Stage, MMSE Mini-Mental State Examination, MSIS-29 Multiple Sclerosis Impact Scale, ng nanogram, OCB oligoclonal bands, SDMT Symbol Digits Modalities Test, SE-ADL Schwab and England Activities of Daily Living scale, VAS Visual Assessment.

**Fig. 2:**
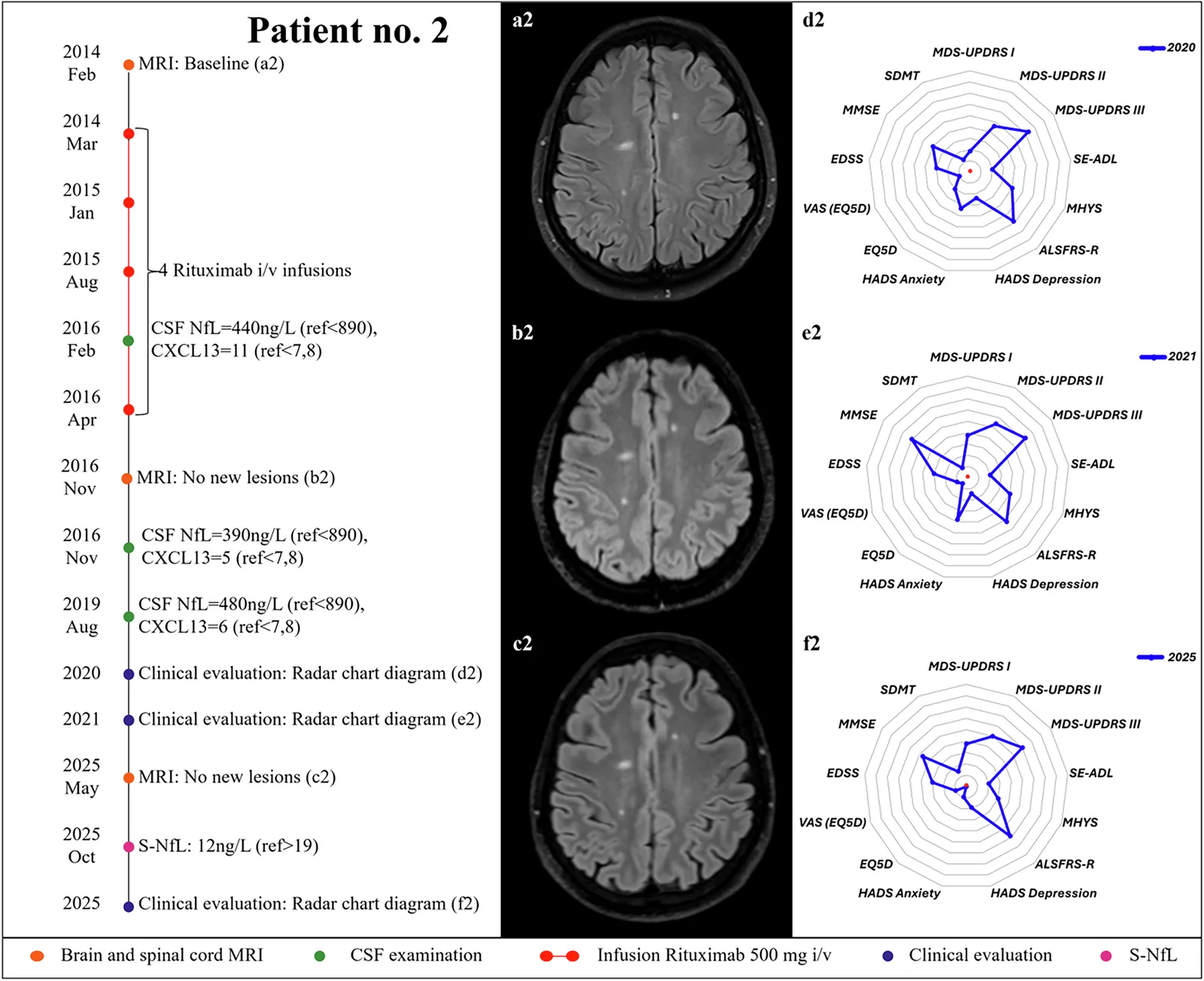
Disease evolution and treatment timelines for patient 2. **a2** first brain MRI (2014); **b2** 2-year follow-up (2016); **c2** 11-year follow-up (2025). Radar chart diagrams with data of multimodal clinical evaluations performed in year **d2** 2020; **e2** 2021; **f2** 2025. Abbreviations: ALSFRS ALS Functional Rating Scale, CXCL Chemokine (C-X-C motif) ligand, EDSS Expanded Disability Status Scale, EQ5D EuroQol-5D standardized instrument for measuring generic health status, HADS Hospital Anxiety Depression Rating Scal, Ig immunoglobulin, IL interleukin, L liter, MDS-UPDRS Movement Disorder Society–Unified Parkinson’s Disease Rating Scale, mg milligram, MHYS Modified Hoehn and Yahr Stage, MMSE Mini-Mental State Examination, MSIS-29 Multiple Sclerosis Impact Scale, ng nanogram, OCB oligoclonal bands, SDMT Symbol Digits Modalities Test, SE-ADL Schwab and England Activities of Daily Living scale, VAS Visual Assessment.

The full longitudinal numerical data for each clinical instrument and time point were presented in Table 3, which constitutes the primary quantitative reference for the clinical assessments. The radar-chart diagrams^22^ in Figs. 1 and 2 provide a complementary visual representation of the multi-domain clinical profile and its evolution across time points.

#### Secondary outcome

Levels of NfL (a biomarker for CNS axonal damage) in CSF and serum were measured as the secondary outcome variable^23^. Serum NfL measurement using single-molecule array (Simoa®) was established for clinical use in Karolinska University Hospital in 2018. Due to this reason, no pre-treatment serum NfL value was available for either patient.

#### Exposure

Patients were given RTX treatment at a dose of 500 mg intravenously every 6 months. RTX was administered off-label and on a compassionate basis. At the time of treatment initiation, no disease-modifying therapy had been established for CRL, and the local multidisciplinary neuroinflammation board reviewed each case individually. The decision to initiate RTX was based on (i) the inflammatory and demyelinating phenotype of brain MRI, which had originally supported a working diagnosis of MS; (ii) elevated CSF biomarkers of B-cell–mediated inflammation, in particular CXCL13; and (iii) emerging evidence in the MS literature supporting B-cell depletion as a therapeutic strategy for inflammatory demyelinating disease. The 500 mg dose every six months followed the established local protocol for secondary-progressive MS. Treatment was planned as a time-limited, exploratory regimen of four infusions, after which clinical, radiological and biomarker responses were reassessed before any decision regarding continuation.

#### Consideration of bias

To reduce potential clinical evaluation bias, patients were examined by VDK together with different co-investigators (Oluf Andersen, ZKW, GCM and MK).

## Methods

### Genetic analysis

The variant in the *CSF1R* gene was confirmed for patients 1 and 2 by Sanger sequencing performed at CENTOGENE GmbH laboratories in Germany. Exon 20 of the *CSF1R* gene was analyzed by PCR and sequencing of both DNA strands and the highly conserved exon-intron splice junctions using the reference sequence NM_005211.3, on a 3730xl sequencer (Thermo Fisher Scientific, Waltham, MA).

The pathogenicity of the identified *CSF1R* sequence variant was classified at CENTOGENE GmbH using their implementation of the ACMG/AMP/ClinGen Sequence Variant Interpretation (SVI) framework^24^, incorporating computational, population-frequency, functional-domain and co-segregation evidence. The applied criteria and the resulting classification are reported in Table 1.

**Table 1:** Demographics and clinical characteristics of patients included in the study

Variable	Patient 1	Patient 2
Age at symptom onset	18–20	40
Clinical symptoms	Weakness in hands, frequent falls	Weakness in hands, memory problems, frequent falls
Sex	Female	Female
Ethno-regional descent	Finnish Kale	Finnish Kale
Gene mutation	Heterozygous *CSF1R* c.2562 T > A,	Heterozygous *CSF1R* c.2562 T > A
Amino acid change	p.Asn854Lys	p.Asn854Lys
Diagnosis according to the diagnostic CRL criteria	Definitive 2a, 2b, 3, 4a	Definitive 2a, 2b, 3, 4a
Pathogenicity classification of sequence variants according to ACMG	Likely pathogenic PM1, PM2_p, PP1_m, PP3_m	Likely pathogenic PM1, PM2_p, PP1_m, PP3_m
RTX administration timepoints	2015–2017	2014–2016
Number of infusions (500 mg)	4	4
Time points of clinical evaluations	2020, 2022, 2025	2020, 2021, 2025
Time points of P-NFL measurement	OCT 2020; FEB, NOV 2021; FEB 2022; APR, OCT 2025	OCT 2020; MAY 2022; OCT 2025

*a* Adenine, *ACMG* American College of Medical Genetics and Genomics, *Arg* Arginine, *Asn* Asparagine, *CSF1R* Colony Stimulating Factor 1 Receptor (NM_005211.3), *Cys* Cysteine, *CRL* *CSF1R*-related leukoencephalopathy, *Lys* Lysine, *mg* Milligram, *N/A* Not Available, PM1 Located in a mutational hotspot or critical functional domain (moderate pathogenic), PM2 absent or extremely rare in population databases (moderate pathogenic, applied at supporting level), P-NFL Neurofilament Light Chain measurement in plasma, PP1 Co-segregation with disease (supporting pathogenic, applied at moderate level), PP3 Computational evidence supports a deleterious effect, REVEL score 0.81 (supporting pathogenic, applied at moderate level), RTX Rituximab, T Thymine, Trp Tryptophan.

### NfL analysis

Concentrations of NfL in CSF and serum were measured using an in-house single-molecule array Simoa® NF-light Advantage Kit (HD-X)^25^.

### Graphical visualization

A spider web diagram, a graphical visualization of clinical evaluations, was performed using Microsoft Excel (2016). A table with clinical evaluations’ raw scores at the three timepoints is presented in Table 3.

### MRI protocol

The standard brain and spinal cord MRIs were conducted according to the protocol described previously^26^. Radiological assessment included three complementary components: (i) qualitative review of focal lesion burden and contrast enhancement on conventional T2-FLAIR and post-contrast T1 sequences; (ii) qualitative assessment of global and regional atrophy, including ventricular and sulcal enlargement; and (iii) quantitative myelin estimation using REMyDI/MyCPF. Rapid Estimation of Myelin for Diagnostic Imaging (REMyDI) and the Quantitative Synthetic MRI analysis of the Myelin Correlated Fraction (MyCPF) were applied for CRL clinical follow-up^27^. REMyDI and MyCPF were originally developed and validated for MS; their application here should be regarded as exploratory rather than a true quantitative measure of treatment response.

### Ethics

The study was approved by both the Regional Ethical Committee in Stockholm, ethical permit Dnr 2009/2017-31/2 “STOPMS-II” and the Swedish Research Ethics Authority, ethical permits Dnr 651-05 “HDLS” and ref. 2019-05742. All patients signed written informed consent to publish the details of health and disease-related information. All procedures were performed in accordance with the principles of the Declaration of Helsinki^28^.

### Data sharing

The data that supports the findings of this study may be available from the corresponding author upon reasonable request and subject to institutional and ethical approvals.

## Results

### Case 1

Patient 1 is the elder of two sisters described in this report; she was the index case in whom the *CSF1R* variant was first identified, leading to subsequent cascade screening of her sister (Patient 2). Patient 1 was initially diagnosed with MS based on the current McDonald diagnostic criteria^29^ in 2002 at an age of 43 years. Over several years the patient received standard immunomodulatory therapies for MS. This included acute relapse management with intravenous methylprednisolone (1 g/day for 3 days in 2002) and disease-modifying therapies during the period 2002–2005: initially Interferon β−1a (30 µg intramuscularly weekly), followed by Interferon β−1a (44 µg subcutaneously three times weekly) (Fig. 1). The latter was discontinued due to the development of high-titer neutralizing antibodies.

According to routine care records, EDSS was 4.5 at CRL diagnosis (2013) and 6.0 at the time of the first RTX infusion (2015), reflecting clinical worsening over the two preceding years. MMSE was not systematically recorded during routine care in this period, and standardized cognitive instruments (MoCA, SDMT) were not part of the routine assessment battery before 2020.

In 2013, Sanger sequencing identified the *CSF1R* missense variant NM_001288705.1:c.2562 T > A, p.Asn854Lys. On reassessment under the ACMG/AMP/ClinGen SVI framework, the variant is classified as likely pathogenic based on computational (PP3_moderate, REVEL 0.81), population-frequency (PM2_supporting), functional-domain (PM1_moderate, tyrosine kinase domain), and co-segregation (PP1_moderate) evidence according to Sundal et al.^12^(Table 1). The diagnostic criteria for definite CRL according to Konno et al.^30^ were fulfilled (Table 1).

Magnetic resonance imaging (MRI) revealed lesions consistent with neuroinflammatory demyelination. Cerebrospinal fluid (CSF)–chemokine (C-X-C motif) ligand 13 (CXCL13) and CSF-NfL were elevated in 2015. By 2015, two years after genetic confirmation of the *CSF1R* variant, the patient continued to show clinical and radiological features compatible with active inflammatory demyelination, including persistently elevated CSF-CXCL13 and CSF-NfL. At this point, the diagnosis was already established as CRL rather than MS; RTX was therefore not initiated under a working MS diagnosis but as an off-label, exploratory intervention specifically intended to address the B-cell–driven inflammatory component superimposed on the underlying *CSF1R*-related microgliopathy. The case was reviewed by the local multidisciplinary neuroinflammation board, which approved a time-limited regimen of four RTX infusions (500 mg every six months) with a planned reassessment thereafter. Treatment was discontinued after four infusions as originally planned. At reassessment, no new MRI lesions were observed, levels of CSF-CXCL13 and CSF-NfL had decreased, and clinical status was stable; in the absence of evidence of ongoing inflammatory activity to justify continued B-cell depletion and given the unknown long-term safety of sustained RTX exposure in a patient with a primary microgliopathy, the multidisciplinary board did not extend treatment beyond the planned four cycles.

**Fig. 3:**
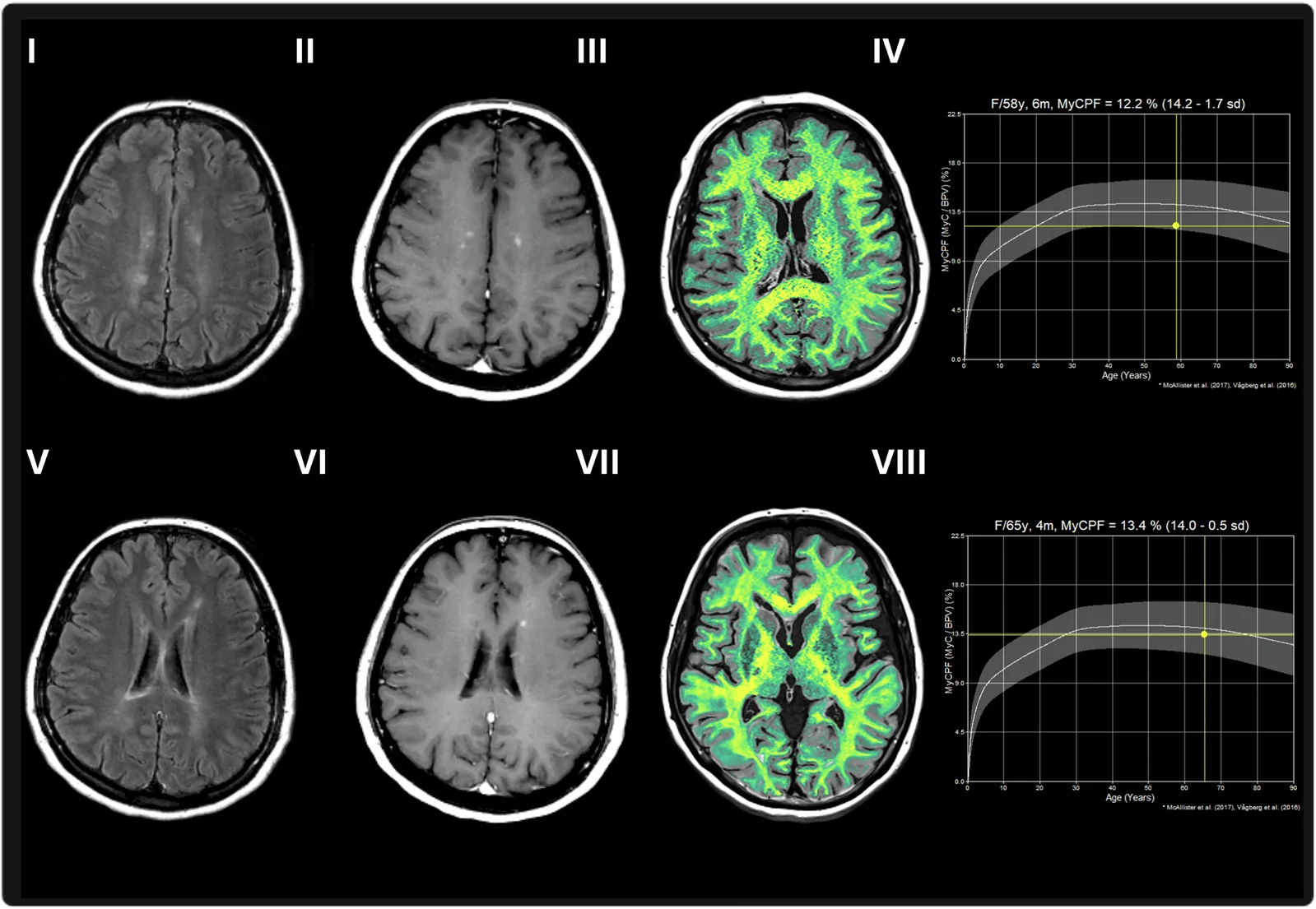
Longitudinal brain MRI with myelin quantification in Patient 1. Brain MRI on the same Siemens Prisma 3 Tesla scanner for Patient 1 at the time point of the last RTX infusion in 2017 (panels I–IV) and in 2024 (7 years after RTX treatment termination, panels V–VIII). I and V correspond to T2-weighted Fluid-Attenuated Inversion Recovery showing multiple periventricular white matter lesions. III and VII correspond to contrast‑enhanced T1-weighted imaging showing small foci of blood-brain-barrier disruption. IV and VIII correspond to myelin quantification maps based on Rapid Estimation of Myelin for Diagnostic Imaging (REMyDI). IV and VIII show the myelin content parenchymal fraction (myelin volume divided by the brain parenchymal volume) of 12.2% at the first time point (−1.7 SD compared to normative data based on age and sex) and 13.4% at the second time point (−0.5 SD); this suggests a cessation of the demyelination rate.

On conventional T2-FLAIR and post-contrast T1 longitudinal imaging sequences, no new focal lesions and no new enhancing lesions were observed between 2017 and the most recent imaging in October 2025 (Fig. 1c1, f1). Follow-up imaging documented widening of cerebrospinal-fluid spaces consistent with progressive cerebral atrophy. Exploratory quantitative myelin estimation using REMyDI showed MyCPF values of 12.2% in 2017 and 13.4% in 2024 (Fig. 3, panels IV and VIII).

CSF-NfL and CSF-CXCL13 levels measured during the RTX cycles were lower than the most recent pre-treatment values (Table 2). Serum NfL was within the reference range at the earliest available measurement (October 2020), approximately three years after the final infusion; pre-treatment and on-treatment serum NfL values were not available.

**Table 2:** Cerebrospinal fluid biomarkers before and after RTX infusion

CSF analysis/ patients	Patient 1		Patient 2		Reference value
Months before/after RTX	Before RTX	Post RTX	Before RTX	Post RTX	-
Year	2015	2017	2012/2013	2016	-
CSF-tau	243	N/A	307	256	<300 ng/L
CSF-beta-amyloid	1190	N/A	1230	1230	>550 ng/L
CSF-phospho-tau	49	N/A	68	58	<60 ng/L
CSF-CXCL13	22	3	N/A	11	<7.8 ng/l
CSF-NFL	1270 §	660 §§	N/A	440 §§	§<890 ng/L §§<660 ng/L
CSF albumin ratio x (10-3)	14.5*	10.8*	7,0**	7,09*	*<9.0 **<7.0
CSF IgG index	0.53*	0.47*	0.87**	0.69*	*<0.7 **<0.63
OCB	Absent	Absent	Present	Present	Absent

*Ig* Immunoglobulin, *IL* Interleukin, *OCB* Oligoclonal bands, *CXCL* Chemokine (C-X-C motif) ligand, *mg*, Milligram, *L* Liter, *ng* Nanogram, *N/A* Not Available, §,§§ CSF-NFL cuf-off value differences due to age, * Reference value from 2015, ** Reference value at 2012.

Across the three standardized follow-up timepoints (2020, 2022, 2025), Patient 1 showed a heterogeneous trajectory rather than a uniform pattern (Fig. 1, Table 3). Disability as measured by EDSS remained at 6.5 across all three timepoints. Schwab and England Activities of Daily Living improved from 70% in 2020 to 40% in 2025 (100% completely independent, 10% completely dependent). Cognitive measures fluctuated rather than declined linearly, with MMSE recorded as 23, 17, and 23 across the three time points and SDMT as 20, 12 and 15, respectively. Conversely, neuropsychiatric scores deteriorated substantially: HADS Depression rose from 5 (2020) to 19 (2025), and HADS Anxiety rose from 7 to 21, both crossing thresholds conventionally considered indicative of clinically significant depression and anxiety. At the most recent assessment in 2025, the patient retained upper limb motor function, intelligible speech, and the ability to operate a motorized wheelchair. The disease was considered stable in motor and global disability domains over five years; neuropsychiatric worsening was caused by adverse external life events.

**Table 3:** Functional estimation scores for patient 1 and patient 2 post RTX treatment assessments in 2020, 2022 and 2025

Variable/Patient	Patient 1			Patient 2		
Year	**2020**	**2022**	**2025**	**2020**	**2021**	**2025**
UPDRS Part I	5.0	8.0	8.0	13.0	10.0	10.0
UPDRS part II	15.0	24.0	16.0	28.0	24.0	26.0
UPDRS part III	17.0	16.0	17.0	40.0	23.0	43.0
UPDRS part V	2.5	4.0	4.0	5.0	5.0	4.0
UPDRS part VI	70.0	50.0	40.0	40.0	40.0	30.0
ALSFRS-R	36.0	29.0	34.0	28.0	25.0	28.0
HADS Depression	5.0	15.0	19.0	16.0	18.0	17.0
HADS Anxiety	7.0	11.0	21.0	14.0	13.0	19.0
EQ5D	10.0	13.0	12.0	12.0	14.0	15.0
VAS (EQ5D)	5.0	2.0	1.0	1.0	1.0	1.0
EDSS	6.5	6.5	6.5	7.0	7.0	7.0
MMSE	23.0	17.0	23.0	12.0	18.0	14.0
SDMT	20.0	12.0	15.0	5.0	4.0	6.0

*UPDRS* Unified Parkinson’s Disease Rating Scale, *ALSFRS* ALS Functional Rating Scale, *HADS* Hospital Anxiety Depression Rating Scale, *EQ5D* EuroQol-5D standardized instrument for measuring generic health status, *VAS* Visual Assessment Scale, *EDSS* Expanded Disability Status Scale, *MMSE* Mini-Mental State Examination, *MSIS-29* Multiple Sclerosis Impact Scale, *SDMT* Symbol Digits Modalities Test, N/A Not Available.

### Case 2

Patient 2 first presented in 2008 with joint pain and fatigue and was diagnosed with seronegative arthritis under rheumatology. She received conventional synthetic disease-modifying anti-rheumatic drugs (csDMARDs) between 2009 and 2014, comprising methotrexate, leflunomide and hydroxychloroquine in sequence or combination, together with intermittent oral corticosteroids. The first neurological symptoms - intermittent upper limb numbness, paresthesia, mild ataxia, and fatigue - emerged during 2010–2011 while the patient was receiving csDMARD therapy. These early symptoms were initially attributed to a rheumatological condition or to its treatment. Progression of sensory–motor deficits, together with new memory complaints, urinary incontinence and balance disturbance prompted neurological referral to Karolinska University Hospital in 2012. Brain and cervical spine MRI demonstrated supratentorial inflammatory white-matter lesions consistent with an MS-like pattern, and a working diagnosis of MS was made.

Following the identification of the *CSF1R* mutation in the patient’s sibling (Patient 1) in 2013, cascade screening was performed. Patient 2 was confirmed to carry the same heterozygous *CSF1R* mutation. By the time of RTX initiation in 2014, csDMARDs were progressively tapered and discontinued. The RTX cycles (2014–2016) were administered without concurrent csDMARD exposure.

Although the working clinical diagnosis in 2012 had been MS, by the time RTX was initiated in 2014 the diagnosis had been revised to CRL based on the cascade-identified *CSF1R* variant. Rituximab was therefore introduced under an established CRL diagnosis as an off-label, exploratory intervention, with the same rationale as for Patient 1. As for Patient 1, treatment was stopped after the fourth infusion according to the pre-specified protocol; at reassessment, CSF-NfL and s-NfL levels were below reference cut-off values, CSF-CXCL13 levels had declined, no new MRI lesions were detected and the clinical course was stable.

From 2014 to 2016 the patient received four cycles of RTX following the same protocol used for Patient 1. Biomarker analysis was conducted to monitor treatment response. After the third infusion, CSF-NfL levels were normal, though CSF-CXCL13 levels remained slightly elevated (Fig. 2). Following the fourth infusion, CSF-NfL levels fell below the laboratory reference cut-off (Table 1, Fig. 2). The earliest available serum NfL measurement (October 2020) was below cut-off, but as with Patient 1, no pre- or on-treatment serum NfL value was available; serum NfL therefore cannot be used to support inference about an immediate treatment effect.

In Patient 2, follow-up imaging in 2016 (Fig. 2b2) and 2025 (Fig. 2c2) revealed no new focal supratentorial inflammatory lesions and no new contrast-enhancing lesions compared with baseline imaging in 2014. As for Patient 1, follow-up imaging nevertheless showed enlargement of cerebrospinal-fluid spaces consistent with progressive cerebral atrophy, indicating ongoing white-matter degeneration despite the absence of new focal lesions. Quantitative myelin estimation was not available for Patient 2.

Across the three standardized follow-up timepoints (2020, 2021, 2025), Patient 2 also showed a heterogeneous course (Table 3, Fig. 2). EDSS remained at 7 throughout. MDS-UPDRS Part I improved from 13 to 10. MDS-UPDRS Part III, in contrast, fluctuated substantially (40 in 2020, 23 in 2021, 43 in 2025) with no evidence of sustained motor improvement. Cognitive measures fluctuated without a directional trend: MMSE 12, 18, 14 and SDMT 5, 4, 6. Neuropsychiatric scores were elevated throughout: HADS Depression score remained relatively stable in the abnormal range (16,18,17) while HADS Anxiety score increased between 2021 and 2025(13–19), crossing the threshold for clinically significant anxiety. The increase in HADS Anxiety in Patient 2 coincided with the same family-level psychosocial events described for Patient 1 - bereavement and a close family member’s health crisis. In the same period, the patient was able to discontinue tramadol and reduce benzodiazepine dosage, which is consistent with reduced symptomatic burden but does not by itself constitute evidence of disease modification. At the most recent follow-up in 2025, the patient maintained partial independence: preserved upper limb dexterity (sufficient for assisted activities of daily living), intelligible speech, and the ability to stand, take a few steps, and operate a motorized wheelchair.

## Discussion

**Fig. 4:**
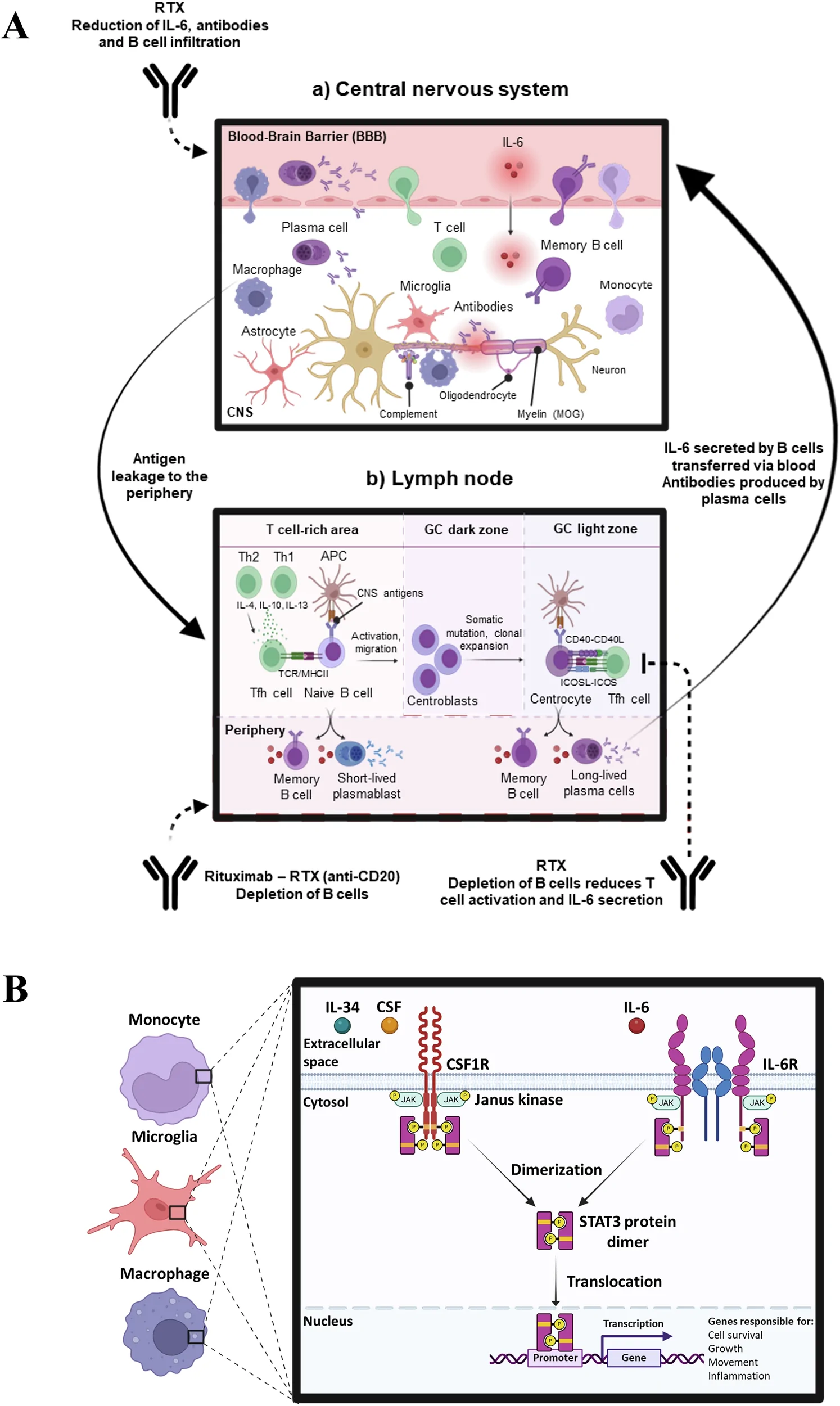
Proposed mechanism of B-cell–mediated neuroinflammation and rituximab action in CSF1R-related leukoencephalopathy. **A** Immune response in leukoencephalopathy: **a** *CSF1R* mutations cause neuroinflammation and blood–brain barrier dysfunction, while rituximab (RTX) eliminates B cells that secrete proinflammatory cytokines and activate inflammatory signaling pathways. **b** Peripheral antigen leakage induces B cell proliferation and differentiation, thereby promoting neuroinflammation, whereas RTX treatment decreases B cell numbers. **B** The STAT3 signaling pathway via CSF1R and IL-6R receptors.

The pathophysiology of CRL is fundamentally driven by microglial dysfunction secondary to haploinsufficiency, resulting in chronic neuroinflammation, white matter degeneration, and a loss of CNS immune homeostasis^11^. Primary CRL pathology is compounded by the infiltration of peripheral CD20⁺ B cells, which secrete IL-6^7^. This cytokine dysregulates the STAT3 signaling axis, shifting it from a regenerative phenotype to a pro-inflammatory state that impedes oligodendrocyte repair^2^ and microglia survival (Fig. 4).

Our mechanistic interpretation is based on a retrospective integration of clinical, radiological, and biomarker data, informed by the observed treatment response, rather than on direct measurement of IL-6, STAT3 phosphorylation, or B-cell phenotype.

Impaired microglial function results in ineffective clearance of cellular debris and myelin breakdown products, fostering a persistent pro-inflammatory milieu within the central nervous system^31^. This environment may contribute to blood–brain barrier dysfunction and facilitate secondary activation of peripheral immune pathways^2,32–34^. As a consequence, CD20⁺ B cells are recruited into the CNS and undergo activation, with antigen-experienced and memory B-cell subsets acting as a significant source of IL-6^7^. Sustained IL-6 production promotes chronic activation of the STAT3 signaling pathway, which, in the context of disrupted *CSF1R*-dependent regulation, becomes proinflammatory rather than reparative^32^. Persistent STAT3 activation is associated with increased oligodendrocyte stress, impaired remyelination, and progressive white matter injury^35^.

The biomarker and clinical patterns observed in these two siblings are compatible with the interruption of a putative IL-6–STAT3 inflammatory loop through CD20⁺ B-cell depletion, though direct mechanistic confirmation would require measurement of B-cell–derived IL-6, STAT3 phosphorylation and microglial activity, which were not assessed in this study. B cell-mediated inflammation may contribute to CRL progression, while B-cell depletion may reduce white matter damage. They do not, in isolation, establish that B-cell–derived inflammation contributes to disease progression in CRL more broadly, nor that B-cell depletion mitigates white matter damage.

### Treatment duration

Both siblings received only four RTX infusions, raising the question of whether prolonged B-cell depletion might have been beneficial. However, several factors informed the decision not to extend treatment: the regimen was predefined as a time-limited exploratory protocol with reassessment after four cycles; follow-up showed reduced biomarkers and no new MRI lesions; the long-term safety of sustained RTX use in primary microgliopathies is unknown; and the regulatory and reimbursement context did not support indefinite off-label use. Thus, whether extended or repeated treatment cycles would be more effective remains an open question for future controlled studies.

During the treatment across both siblings, the standardized scales demonstrated a heterogeneous picture: stability in disability (EDSS) and selected motor domains, fluctuation in motor scores particularly sensitive to short-term state (MDS-UPDRS Part III), variability rather than consistent change in cognitive measures (MMSE, SDMT), and clear worsening of neuropsychiatric domains (HADS) in Patient 1, and a late rise in HADS-Anxiety in Patient 2. We restrict claims of stability or improvement to specific, named domains rather than to the global clinical state.

### Interpretation of HADS scores in the context of psychosocial factors, CRL activity and RTX treatment

HADS is a screening instrument sensitive to acute and subacute psychosocial stressors as well as to underlying mood disorders; during the follow-up period both patients experienced identifiable life events in addition to the cumulative psychosocial burden of progressive physical disability. Available evidence indicates that anti-CD20 therapy is not expected to exert a primary anxiolytic or antidepressant effect^36^. The absence of attenuation in HADS scores following RTX is therefore consistent with the known pharmacological profile of the agent rather than being indicative of treatment failure with respect to the underlying disease process.

### Imaging biomarkers

In a primary microgliopathy with diffuse white-matter degeneration, the absence of new focal T2 lesions and the absence of new contrast-enhancing lesions are necessary but not sufficient conditions for radiological stability. Both siblings showed enlargement of cerebrospinal-fluid spaces consistent with progressive cerebral atrophy on long-term follow-up, which is the expected imaging correlate of continuing diffuse parenchymal loss. We therefore restrict the radiological observations to two specific findings: (i) no new focal or enhancing lesions on conventional sequences over a follow-up period of 8–11 years after the final RTX infusion, and (ii) the persistence of an inflammatory-demyelinating pattern that did not progress to confluent new lesion formation. Whether either of these observations reflects a treatment effect, the natural history of the c.2562 T > A variant, or unmeasured factors cannot be determined in an uncontrolled retrospective design without a comparator cohort. The use of MyCPF data in current work is methodological proof-of-concept for the application of quantitative myelin imaging in CRL.

The strength of this study lies in the biological rationale supporting the exploration of RTX in CRL. Impaired STAT3 signaling reduces microglial numbers and limits their capacity to clear debris, resulting in persistent CNS inflammation and progressive tissue damage^37,38^. This is further compounded by activation of peripheral CD20⁺ B cells, which migrate into the CNS and secrete interleukin-6 (IL-6), thereby amplifying inflammatory signaling and reinforcing STAT3 dysregulation in a self-sustaining cycle that drives white-matter injury^32,33,39^. Under physiological conditions, CSF1R signaling maintains microglial homeostasis through PI3K–AKT and MAPK pathways, whereas its dysfunction promotes neurodegeneration, demyelination, and a shift toward cytokine-mediated inflammatory states^40^. In this context, the IL-6–STAT3 pathway represents a key axis of reactive glial activation, in which IL-6–producing astrocytes, microglia, and peripheral immune cells activate JAK kinases, leading to STAT3 phosphorylation, its movement into the nucleus, and changes in gene expression that promote astrogliosis and astrocytic phenotypic alteration^41^. In parallel, the FGF–FGFR–STAT3 axis functions as a partially compensatory growth factor pathway, where FGFR activation triggers MAPK/ERK and PI3K signaling and contributes to STAT3 activation through JAK-mediated signaling, thereby supporting glial proliferation and tissue repair^42^. In the setting of CSF1R dysfunction, the CNS transitions into an alternative activation state in which the IL-6–STAT3 and FGF–FGFR–STAT3 pathways act both as compensatory responses and drivers of disease progression, contributing to increased astrocyte reactivity, sustained inflammatory signaling, and ongoing white-matter damage^43^. By targeting CSF1R-associated dysregulation through mechanisms involving the FGF–FGFR–STAT3 axis, the therapeutic approach is grounded in mechanistic insights recently highlighted by Du et al.^2^. To our knowledge, this is the first clinical report describing RTX exposure in patients with genetically confirmed CRL, with detailed long-term clinical and biomarker follow-up. For the first time, to the best of our knowledge, the Quantitative Synthetic MRI analysis of the Myelin Correlated Fraction was applied as exploratory measure to evaluate disease progression and treatment outcome in a CRL patient (Fig. 3). This technique was originally developed for longitudinal monitoring in multiple sclerosis^27^.

### Limitations

The most important limiting factor of this study is that it concerns a small and highly selected cohort of only two individuals with genetically confirmed CRL. Critically, both patients are siblings carrying the same heterozygous *CSF1R* variant (c.2562 T > A, p.Asn854Lys). The shared genetic background, environmental exposures and disease trajectory-limited to this specific variant within a single family thus reduce generalization of the clinical observations. In particular, the heterogeneous and prolonged disease course before RTX in both siblings confounds our conclusions. Patient 1 had experienced over a decade of symptomatic disease before treatment and a total duration of approximately 23 years by 2025, while CRL typically has a median survival of around 8 years, with only a minority exceeding 20 years. She likely represents a slow-progressing phenotype independent of treatment and her post-treatment stability is compatible with, but does not require, any effect of RTX. In contrast, Patient 2 had a shorter pre-treatment course, with symptom onset around age 40 and treatment initiation about two years later, limiting assessment of untreated progression and interpretation of treatment effects.

### A related confounder is prior immunomodulatory treatment in both patients, precluding a truly treatment-naïve baseline

Patient 1 had received interferon β-1a, and Patient 2 had received methotrexate, leflunomide and hydroxychloroquine, all of which can potentially affect immune pathways. These prior therapies may have influenced disease course, so post-RTX effects cannot be interpreted independently of earlier immunomodulation or potential carry-over effects.

### All of these confounders are compounded by the lack of an identical assessment battery pre–post treatment

Standardized assessments only began in 2020, several years after the last RTX infusion, with no consistent prior data. Moreover, the clinical instruments used (MDS-UPDRS, ALSFRS-R, EDSS, MMSE, SDMT, HADS, MSIS-29, EQ-5D) have not been validated for CRL, and minimal clinically important differences in this population remain unknown. In addition, the biomarker dataset is not fully symmetrical across the two patients, with Patient 1 having both pre- and post-treatment CSF measurements, and Patient 2 having on-treatment but not pre-treatment values. Additionally, mechanistic evidence is lacking as IL-6, STAT3 signaling and B-cell phenotyping were not assessed. The proposed mechanism therefore remains hypothetical and requires validation in future studies.

### Furthermore, there are limitations in imaging assessments

Firstly, the absence of new focal lesions is not a sufficient marker of radiological stability in a diffuse leukoencephalopathy, and quantitative volumetric measures of atrophy and white-matter integrity were not systematically applied across both patients and at all time points. Secondly, although a promising quantitative myelin technique^27^, REMyDI/MyCPF has not been validated in CRL; its application here is methodological and exploratory, and the values reported should not be used to infer treatment response.

Finally, the classification of the *CSF1R* p.Asn854Lys variant as likely being pathogenic relies on the ACMG/AMP/ClinGen SVI framework as implemented in our reference genetics laboratory, drawing on computational, population-frequency, functional-domain and co-segregation evidence rather than on PS1, which is not applicable in the absence of an alternative pathogenic substitution at codon 854 in the published literature or in ClinVar. However, variant classification in ultra-rare neurodegenerative disorders such as CRL is inherently constrained by the small number of reported variants at any given residue, and a classification of variant of uncertain significance (VUS) under a more stringent application of the criteria would not be unreasonable. Importantly, this consideration does not alter the diagnostic basis for inclusion of the two siblings in the present report: both patients fulfill the Konno *et al*. (2018) criteria for definite CRL^30^.

### Patients’ perspective

CRL is a rare disease with limited choices of effective treatment to halt disease progression. Patients endorsed the publication about the disease evolution and well-tolerated RTX treatment to disseminate knowledge to other patients, patient organizations, and professionals.

## Conclusions

This preliminary familial case series describes the first reported RTX exposure in two siblings with genetically confirmed CRL. Rituximab exposure was followed by long-term clinical stability in EDSS, reductions in levels of CSF-CXCL13 and CSF-NfL between pre-treatment and on-treatment timepoints, and absence of new focal lesions on follow-up brain MRI in the presence of ongoing parenchymal atrophy. However, our observations should be interpreted in the context of an inherently slowly progressive disease course of more than a decade before RTX initiation, which cannot be entirely disentangled from the observed pharmacological effects. Our observations are compatible with, but do not establish, modulation of downstream inflammatory pathways. While these findings do not confirm RTX as a disease-modifying therapy in CRL, they do showcase a likely potential of targeting IL-6-secreting B cells as a novel therapeutic approach for the management of *C*RL. Prospective, controlled studies, including comparison with natural-history cohorts matched for variant and pre-treatment disease duration, will be required to evaluate whether B-cell–directed strategies have a measurable effect on disease trajectory in CRL.

## Supplementary information

Transparent Peer Review file

## Data Availability

All data generated or analysed during this study are included in this published article.
