# Supplementary material for: Long-term clinical and biomarker observations following rituximab exposure in two siblings with CSF1R-related leukoencephalopathy: a preliminary familial case series
Source: Commun Med (Lond). 2026 Sep 30;6:514. doi: 10.1038/s43856-026-01936-5 (PMC13633238; doi:10.1038/s43856-026-01936-5)
Supplement: Supplementary file 1 [file 43856_2026_1936_MOESM1_ESM.pdf]

nature portfolio

Peer Review File

Long-term clinical and biomarker observations following rituximab exposure in two siblings with CSF1R-related leukoencephalopathy: a preliminary familial case series

Corresponding Author: Dr Virginija Karrenbauer

This file contains all reviewer reports in order by version, followed by all author rebuttals in order by version.

Version 0:

Reviewer comments:

Reviewer #1

(Remarks to the Author)
Brief Summary: This manuscript reports the clinical outcomes of two genetically confirmed CSF1R-related leukoencephalopathy (CRL) patients treated with rituximab (RTX), a CD20⁺ B-cell–depleting monoclonal antibody. Both patients, who are siblings carrying the same heterozygous CSF1R c.2562T>A (p.Asn854Lys) variant, received four infusions of RTX at 500 mg. The authors report clinical stabilization, normalization of CSF and serum biomarkers, and absence of new MRI lesions over a follow-up period extending to 2025. The authors propose a mechanistic model in which B-cell–derived IL-6 perpetuates STAT3 dysregulation and white matter injury, and argue that RTX may interrupt this inflammatory cycle.

Overall Impressions: This is an interesting case report describing the first use of RTX in CRL with long-term follow-up. The biological rationale linking B-cell–derived IL-6 to STAT3-mediated pathology is thoughtful and well-articulated. The long follow-up period is a strength. However, several issues regarding nomenclature, variant classification, and interpretation of clinical course require attention before this manuscript is suitable for publication.

Specific comments:
1. Nomenclature typo (line 61): "CSF1R-related encephalopathy (CRL)" should be corrected to "CSF1R-related leukoencephalopathy (CRL)" to remain consistent with the rest of the manuscript and established nomenclature.
2. Please italicize genes throughout the manuscript (e.g. CSF1R, lines 85 and elsewhere), in accordance with standard genetic nomenclature conventions.
3. ACMG classification - PS1 criterion (Table 1): The authors classify the p.Asn854Lys variant as "Likely pathogenic" using ACMG criteria PS1 and PM1. However, PS1 requires that the same amino acid change has been previously established as pathogenic regardless of nucleotide change. To my knowledge, p.Asn854Lys is the only CRL-associated variant at amino acid position 854. There are no other reported pathogenic variants resulting in a different amino acid change at this position in the published literature or on ClinVar. The authors should revisit whether PS1 is appropriately applied here, and if not, reassess the variant classification accordingly. Even if the variant were reclassified as a VUS, this would not substantially alter the conclusions of the paper or the justification for including these patients, as both meet the Konno et al. diagnostic criteria for definite CRL. The VUS designation would simply reflect the inherent limitation of variant classification in ultra-rare diseases, and this should be acknowledged.
4. Disease duration prior to RTX in Patient 1: Patient 1 first developed symptoms in or prior to 2002 (first received methylplrednisolone for MS in 2002) and did not receive rituximab until 2015, representing approximately 13 years of disease prior to treatment initiation. Given that the average disease duration from diagnosis to death in CRL is roughly 8 years, this patient appears to have had an unusually mild or slowly progressive disease course independent of RTX. This is an important confounder, as the observed clinical stability post-RTX could partly reflect the patient's inherently indolent disease trajectory rather than a treatment effect. The authors should discuss this limitation explicitly.
5. Patient 2 timeline clarification: The age at symptom onset for Patient 2 is listed as 40 (Table 1), and MS was suspected in 2012. The patient received various DMARDs for seronegative arthritis between 2009–2014. Please clarify the temporal relationship between symptom onset, the MS (mis)diagnosis, and the initiation of immunomodulatory therapies. Were any of the neurological symptoms present before or concurrent with the arthritis treatment period? Were any of the DMARDs potentially modifying the neurological disease course prior to RTX? This is relevant to interpreting the baseline disease

trajectory.
6. Table 3: The HADS-anxiety scores increased significantly for both patients between 2020-2025, and the HADS-depression scores increased significantly for patient 1 while remaining stable for patient 2 between 2020-2025. Please comment on this trend. While other reported measures in this manuscript are certainly consistent with clinical stabilization, these scores make the stabilization narrative a bit more nuanced.

Reviewer #2

(Remarks to the Author)
Summary
This manuscript presents a case series of two siblings from the same family, both with genetically confirmed CSF1R-related leukoencephalopathy due to the same heterozygous CSF1R variant, who received rituximab and underwent long-term clinical, biomarker, and MRI follow-up.

Overall assessment
Given the rarity of this disorder and the scarcity of therapeutic data, the longitudinal follow-up in these cases is valuable. However, as this is a retrospective observational report involving two related individuals from a single family, and as the available clinical, biomarker, and imaging data are limited, the manuscript appears to overstate both the causal relationship between rituximab treatment and the observed clinical course and the mechanistic implications of the findings. The data are more appropriately interpreted as a descriptive and exploratory case series rather than evidence of disease modification by rituximab. Major revision is therefore warranted.

Major comments

1. Claims regarding the therapeutic effect of rituximab are overstated.
The central concern is that the manuscript overinterprets the therapeutic effect of rituximab in CSF1R-related leukoencephalopathy. Given that this is a retrospective observational report of only two related individuals from a single family, the findings are more appropriately presented as a preliminary familial case series. The overall framing should therefore be moderated. As written, several statements are stronger than the data can support.

2. The familial nature of the case series should be made clear earlier.
It should be stated from the outset that the report concerns two siblings from the same family carrying the same heterozygous CSF1R variant. At present, this only becomes fully apparent when the reader reaches the description of Case 2. This point deserves explicit mention in the Abstract and early in the main text, because it affects how the observations are interpreted, including the apparent treatment effect and the extent to which the findings can be generalized.

3. The treatment rationale and the reason for discontinuing rituximab should be clarified.
Because rituximab is not an established treatment for CSF1R-related leukoencephalopathy, the clinical rationale for its use in each case needs to be described more clearly. In particular, it is important to clarify whether rituximab was given under a working diagnosis of multiple sclerosis, or whether it was introduced after recognition of CSF1R-related disease as an off-label, exploratory approach. The reason for stopping treatment after only four infusions should also be stated explicitly. If the authors regard rituximab as beneficial, readers will reasonably ask why it was not continued. Indicating whether discontinuation was planned from the outset or influenced by efficacy, safety, tolerability, or practical considerations would help place the subsequent course in proper context.

4. The assessment timeline does not allow a meaningful pre-/post-treatment comparison.
A key limitation is that the standardized follow-up assessments were performed several years after rituximab exposure rather than before and after treatment. Rituximab was administered in 2015–2017 in Case 1 and 2014–2016 in Case 2, whereas much of the reported clinical and biomarker follow-up appears to begin only in 2020. As presented, this does not provide a meaningful pre-/post-treatment comparison and therefore substantially limits inference regarding treatment effect. In particular, statements implying that normalization of serum NfL reflects the effect of rituximab should be made more cautiously, since serum NfL was measured only years later. This limitation should be acknowledged explicitly. In addition, the report would be strengthened by clearer presentation of any baseline clinical data available from routine care. Baseline EDSS and cognitive measures such as MMSE would be especially informative for interpreting the later follow-up data.

5. The interpretation of the clinical assessment results should be more cautious.
A clearer explanation of why these particular clinical scales were chosen would be helpful. Although several of the selected measures are understandable in this disease context, the rationale for the full assessment battery is not fully explained. More importantly, the reported scores vary considerably across follow-up, making claims of “stability” or “improvement” difficult to evaluate. For example, HADS scores in Case 1 worsen over time, UPDRS Part III in Case 2 fluctuates substantially, and MMSE changes markedly in both cases. Taken together, these data do not show a consistent pattern of clinical improvement. If the authors wish to retain such claims, they should specify which domains appeared to improve or remain stable, and explain why those changes should be considered clinically meaningful.

6. The MRI findings are overinterpreted.
The imaging results are interpreted too strongly in support of disease modification. In a progressive leukoencephalopathy such as CSF1R-related leukoencephalopathy, the absence of new focal lesions alone is not sufficient to infer radiological stabilization. Greater emphasis should be placed on atrophy and diffuse white matter degeneration. Notably, the manuscript itself describes enlargement of CSF spaces consistent with atrophy in Case 1, which seems difficult to reconcile with a

strong claim of imaging stability. In addition, although REMyDI and MyCPF are interesting exploratory measures, their use as markers of disease progression or treatment response in CRL is not yet sufficiently justified here. A more balanced discussion of what the MRI findings do and do not support would strengthen this section.

7. The proposed mechanism is insufficiently supported by the available data.
The Discussion links the apparent effect of rituximab to depletion of IL-6-producing CD20-positive B cells and disruption of an IL-6–STAT3 feedback loop. However, the current data do not directly support this mechanistic interpretation. Neither IL-6 nor STAT3-related markers were measured, and no B-cell phenotyping data are provided. Although NfL and CXCL13 were assessed, these biomarkers are not sufficient to substantiate the proposed pathway. In addition, the biomarker data are not presented in a fully comparable manner across the two cases: Case 1 includes pre- and post-treatment measurements, whereas the corresponding baseline data for Case 2 appear to be unavailable in Table 2, and the values described in Figure 3 seem to have been obtained during treatment rather than before treatment. For this reason, the mechanistic discussion would be more appropriate as a hypothesis than as a conclusion supported by the present report.

Minor comments

1. Please clarify which parkinsonism rating scale was used.
The manuscript refers to “UPDRS I–VI.” If the original UPDRS was used, this should be stated clearly and cited appropriately, with a brief justification for its use. Otherwise, the terminology should be revised to MDS-UPDRS, for which only Parts I–IV apply.

2. The reference to Table 2 at line 172 may be incorrect.
Table 2 presents CSF biomarker data and does not seem to correspond to the content described in the text. Please check whether a different table was intended.

3. The timing of the clinical assessments in Figure 3 should be checked for consistency with the text and Table 3.
The legend for Figure 3 indicates that the clinical assessments in Case 2 were performed in 2020, 2021, and 2025, whereas the main text and Table 3 indicate 2020, 2022, and 2025. Please verify and correct these time points as needed.

4. The radar charts may not be the most suitable format for presenting the primary longitudinal data.
In a case series of only two patients, a simple longitudinal table may be easier to interpret than a radar chart.

Version 1:

Reviewer comments:

Reviewer #1

(Remarks to the Author)
Brief Summary: This manuscript reports the clinical outcomes of two genetically confirmed CSF1R-related leukoencephalopathy (CRL) patients treated with rituximab (RTX), a $CD20^+$ B-cell–depleting monoclonal antibody. Both patients, who are siblings carrying the same heterozygous CSF1R c.2562T>A (p.Asn854Lys) variant, received four infusions of RTX at 500 mg. The authors report a heterogeneous clinical trajectory that included stabilization of some clinical measures. The authors propose a mechanistic model in which B-cell–derived IL-6 perpetuates STAT3 dysregulation and white matter injury, and argue that RTX may interrupt this inflammatory cycle.

Overall Impression: The authors' revision satisfactorily addresses most concerns that were brought up during the initial review. I recommend acceptance with only a few minor changes.

1. Various words do not need capitalization (multiple sclerosis, rituximab, etc.)
2. Line 37 typo: "L" in "Long-term" should be lowercase
3. Please ensure all mentions of the gene CSF1R are italicized, while all mentions of the protein CSF1R are not italicized. Most of these are now correct; in line 431, please italicize "CSF1R."
4. Lines 485-486: "CSF1R-relative neurodegeneration" - if referring specifically to CRL, recommend using the term "CRL" to maintain consistency. Otherwise, please elaborate on difference between CSF1R-related neurodegeneration vs CRL.

Reviewer #2

(Remarks to the Author)
The authors have adequately addressed my major concerns through substantial revisions. In particular, the revised manuscript now clearly acknowledges the limitations of the retrospective familial case-series design, the lack of a formal pre-/post-treatment comparison, the heterogeneous clinical trajectories, the ongoing cerebral atrophy, and the absence of direct evidence for the proposed mechanism. The rationale for rituximab treatment and the decision to limit treatment to four infusions have also been clarified. Overall, the revised manuscript is substantially more balanced and appropriately distinguishes the reported observations from hypothesis-generating interpretations. I have no further comments.

Open Access This Peer Review File is licensed under a Creative Commons Attribution 4.0 International License, which permits use, sharing, adaptation, distribution and reproduction in any medium or format, as long as you give appropriate credit to the original author(s) and the source, provide a link to the Creative Commons license, and indicate if changes were made.
In cases where reviewers are anonymous, credit should be given to 'Anonymous Referee' and the source.
The images or other third party material in this Peer Review File are included in the article's Creative Commons license, unless indicated otherwise in a credit line to the material. If material is not included in the article's Creative Commons license and your intended use is not permitted by statutory regulation or exceeds the permitted use, you will need to obtain permission directly from the copyright holder.
To view a copy of this license, visit https://creativecommons.org/licenses/by/4.0/

Point-by-point response to Reviewers

Manuscript: "Long-term clinical and biomarker observations following rituximab exposure in two siblings with CSF1R-related leukoencephalopathy: a preliminary familial case series". Line numbers refer to the revised manuscript (continuous line numbering, left margin). Text shown in blue in the revised manuscript file corresponds to content added or modified in response to the reviewers' comments summarised below.

	Answers, explanations, text amendments
Referee #1: clinical neurology, gene therapy and therapeutic development for leukoencephalopathy, efficacy and toxicity testing<br>Referee #2: neurology, gene therapy and therapeutic development for leukoencephalopathy	We thank both referees for their expert and constructive evaluation of our manuscript. Their combined expertise in clinical neurology, genetics and therapeutic development for leukoencephalopathies has substantially strengthened the rigour, transparency and balance of the revised manuscript. A point-by-point response to each comment is provided below; all corresponding text amendments are shown in **blue** in the revised manuscript file, and are referenced by their current line number (continuous numbering, left margin).
**Reviewer 1**	
Brief Summary: This manuscript reports the clinical outcomes of two genetically confirmed CSF1R-related leukoencephalopathy (CRL) patients treated with rituximab (RTX), a CD20^{+} B-cell–depleting monoclonal antibody. Both patients, who are siblings carrying the same heterozygous CSF1R c.2562T>A (p.Asn854Lys) variant, received four infusions of RTX at 500 mg. The authors report clinical stabilization, normalization of CSF and serum biomarkers, and absence of new MRI lesions over a follow-up period extending to 2025. The authors propose a mechanistic model in which B-cell–derived IL-6 perpetuates STAT3 dysregulation and white matter injury, and argue that RTX may interrupt this inflammatory cycle.<br>Overall Impressions: This is an interesting case report describing the first use of RTX in CRL with long-term follow-up. The biological rationale linking B-cell–derived IL-6 to STAT3-mediated pathology is thoughtful and well-articulated. The long follow-up period is a strength. However, several issues regarding nomenclature, variant classification, and interpretation of clinical course require attention before this manuscript is suitable for publication.	We thank Reviewer 1 for this positive overall assessment and for identifying the specific issues of nomenclature, variant classification and interpretation that required attention. Each point is addressed individually below (R1.1–R1.6); collectively, these revisions have led to a more precise manuscript.
1. Nomenclature typo (line 61): "CSF1R-related encephalopathy (CRL)" should be corrected to "CSF1R-related leukoencephalopathy (CRL)" to remain consistent with the rest of the manuscript and established nomenclature.	**R1.1.** We thank the reviewer for identifying this inconsistency. We have systematically re-checked every occurrence of the disease term throughout the manuscript to ensure that the established nomenclature "*CSF1R*-related leukoencephalopathy (CRL)" is used consistently, including in the Plain Language Summary, where the shortened, incorrect form had been used. The corrected term now appears consistently from first use onward, including in the Abstract Background (line 28), the Plain Language Summary (line 52), and the Introduction (line 65), and throughout the Methods, Results and Discussion. This correction is coordinated with the revised Title (R2.2), which also uses the full, corrected disease term.

	Answers, explanations, text amendments
2. Please italicize genes throughout the manuscript (e.g. CSF1R, lines 85 and elsewhere), in accordance with standard genetic nomenclature conventions.	**R1.2.** We agree that gene symbols should be italicised throughout, in accordance with HUGO Gene Nomenclature Committee (HGNC) conventions, while the corresponding protein products are retained in non-italic (Roman) type. The original manuscript was inconsistent in this respect. We have systematically revised the manuscript so that all gene symbols (*CSF1R*, *STAT3*, *CSF1*, *IL6*, *IL34*) are italicised — in the Title, Abstract, Plain Language Summary, Introduction (lines 65–115), Patients & Methods, Results (e.g. line 214, line 227, line 308), Discussion (lines 345–423) and Conclusions (lines 476–489) — while protein products (CSF1R receptor, STAT3, IL-6) and HGVS variant nomenclature (c.2562T>A, p.Asn854Lys) remain in Roman type, and Tables 1–3 and the Figure 4 legend have been corrected accordingly.
3. ACMG classification - PS1 criterion (Table 1): The authors classify the p.Asn854Lys variant as "Likely pathogenic" using ACMG criteria PS1 and PM1. However, PS1 requires that the same amino acid change has been previously established as pathogenic regardless of nucleotide change. To my knowledge, p.Asn854Lys is the only CRL-associated variant at amino acid position 854. There are no other reported pathogenic variants resulting in a different amino acid change at this position in the published literature or on ClinVar. The authors should revisit whether PS1 is appropriately applied here, and if not, reassess the variant classification accordingly. Even if the variant were reclassified as a VUS, this would not substantially alter the conclusions of the paper or the justification for including these patients, as both meet the Konno et al. diagnostic criteria for definite CRL. The VUS designation would simply reflect the inherent limitation of variant classification in ultra-rare diseases, and this should be acknowledged.	**R1.3.** We thank the reviewer for this expert and entirely correct observation: PS1 requires that the *same amino-acid change* has previously been established as pathogenic irrespective of the underlying nucleotide substitution, and to our knowledge no other pathogenic variant at codon 854 of *CSF1R* has been reported in the literature or in ClinVar. In response, our co-author and clinical geneticist (N.Z., CENTOGENE GmbH) has re-reviewed the variant using CENTOGENE's implementation of the ACMG/AMP/ClinGen Sequence Variant Interpretation (SVI) framework. PS1 and PM1 have been removed from the original assessment. The variant *CSF1R* NM_001288705.1:c.2562T>A, p.Asn854Lys retains a classification of **likely pathogenic** under the revised evidence combination — PP3_moderate (REVEL 0.81), PM2_supporting, PM1_moderate (tyrosine-kinase domain) and PP1_moderate (co-segregation) — as now reported in the Methods (lines 182–185) and Results (lines 227–232) and in the revised Table 1 (lines 233–241). We have also added an explicit acknowledgment in the Limitations (lines 462–471) that variant classification in ultra-rare disorders such as CRL is inherently constrained by the small number of reported variants at any given residue, that a VUS classification under a stricter reading would not be unreasonable, and that this does not affect the diagnostic basis for inclusion of both siblings, who fulfil the Konno et al. (2018) criteria for definite CRL independently of the ACMG call.
4. Disease duration prior to RTX in Patient 1: Patient 1 first developed symptoms in or prior to 2002 (first received methylplrednisolone for MS in 2002) and did not receive rituximab until 2015, representing approximately 13 years of disease prior to treatment initiation. Given that the average disease duration from diagnosis to death in CRL is roughly 8 years, this patient appears to have had an unusually mild or slowly progressive disease course independent of RTX. This is an important confounder, as the observed clinical stability post-RTX could partly reflect the patient's inherently indolent disease trajectory rather than a treatment effect. The authors should discuss this limitation explicitly.	**R1.4.** We agree, and thank the reviewer for highlighting a confounder that materially affects the interpretation of Patient 1's clinical course. Symptom onset occurred at age 18–20 and the first treated relapse dates to 2002; rituximab was initiated only in 2015, i.e. after approximately 13 years of symptomatic disease, against a reported median survival from onset of roughly 8 years in CRL. This has been made explicit as a new paragraph in the Discussion (lines 430–441 of the Limitations section, which we have consolidated with the Discussion for this point), stating that Patient 1's pre-treatment course places her toward the slowly progressive end of the CRL spectrum independent of rituximab, and that the post-2020 clinical stability is compatible with, but does not require, a treatment effect. The same caveat, applied proportionately to Patient 2's shorter (~2-year) pre-treatment interval, is now stated at lines 439–441. This confounder is carried through to the Abstract Conclusions (lines 48–50) and to the manuscript Conclusions (lines 481–483), which now explicitly state that the observed long-term stability of Patient 1 cannot be disentangled from her inherently slow disease trajectory.
5. Patient 2 timeline clarification: The age at symptom onset for Patient 2 is listed as 40 (Table 1), and MS was suspected in 2012. The patient received various DMARDs for seronegative arthritis between 2009–2014. Please clarify the temporal relationship between symptom onset, the MS (mis)diagnosis, and the initiation of immunomodulatory therapies. Were any of the neurological	**R1.5.** We thank the reviewer for this important request for chronological clarity. We have re-examined the clinical record and revised the Case 2 narrative (lines 296–317) to state explicitly that Patient 2 first presented in 2008 with joint pain and fatigue and was diagnosed with seronegative arthritis; that conventional synthetic DMARDs (methotrexate, leflunomide, hydroxychloroquine, with intermittent corticosteroids) were given between 2009 and 2014; that the first neurological symptoms (intermittent upper-limb numbness, paraesthesia,

	Answers, explanations, text amendments
symptoms present before or concurrent with the arthritis treatment period? Were any of the DMARDs potentially modifying the neurological disease course prior to RTX? This is relevant to interpreting the baseline disease trajectory.	mild ataxia, fatigue) emerged in 2010–2011, i.e. **while** the patient was already receiving csDMARD therapy for arthritis; and that neurological referral, brain/spine MRI and a working MS diagnosis followed in 2012, with cascade genetic screening and diagnostic revision to CRL in 2013–2014. csDMARDs were tapered and discontinued by the time rituximab was started in 2014, so the rituximab cycles (2014–2016) were not confounded by concurrent csDMARD exposure (line 309–310). We have added a dedicated paragraph to the Discussion/Limitations (lines 442–446) noting that methotrexate, leflunomide (the parent compound of teriflunomide, an approved MS disease-modifying therapy) and hydroxychloroquine each have immunomodulatory activity that could plausibly have influenced the neuroinflammatory component of Patient 2's disease prior to rituximab, so that her pre-rituximab baseline cannot be regarded as treatment-naive; this is now also captured in Table 1 as a new "prior immunomodulatory therapy" entry.
6. Table 3: The HADS-anxiety scores increased significantly for both patients between 2020-2025, and the HADS-depression scores increased significantly for patient 1 while remaining stable for patient 2 between 2020-2025. Please comment on this trend. While other reported measures in this manuscript are certainly consistent with clinical stabilization, these scores make the stabilization narrative a bit more nuanced.	**R1.6.** We agree that the HADS trajectory (Depression 5→15→19 and Anxiety 7→11→21 in Patient 1; Depression 16→18→17 and Anxiety 14→13→19 in Patient 2, crossing conventional thresholds for clinically significant symptoms) is not compatible with a uniform narrative of stabilisation and required explicit comment rather than being absorbed into the overall clinical summary. We have revised the Case 1 (lines 273–284) and Case 2 (lines 331–341) narratives to report the HADS trend transparently, and have added a dedicated paragraph in the Discussion ("Interpretation of HADS Scores…", lines 384–391) attributing the deterioration principally to documented psychosocial stressors occurring during the follow-up window — the COVID-19 pandemic and associated isolation, a bereavement within the immediate family, and a close family member's health crisis — superimposed on the cumulative psychosocial burden of progressive physical disability. We note that anti-CD20 therapy is not expected to exert a primary anxiolytic or antidepressant effect, so that the absence of HADS improvement is consistent with the known pharmacological profile of rituximab rather than indicative of treatment failure with respect to the underlying neurological disease. This nuance is now reflected in the Abstract Results (lines 41–44) and the Limitations (consolidated with lines 447–455).
**Reviewer 2**	
Summary This manuscript presents a case series of two siblings from the same family, both with genetically confirmed CSF1R-related leukoencephalopathy due to the same heterozygous CSF1R variant, who received rituximab and underwent long-term clinical, biomarker, and MRI follow-up.<br><br>Overall assessment Given the rarity of this disorder and the scarcity of therapeutic data, the longitudinal follow-up in these cases is valuable. However, as this is a retrospective observational report involving two related individuals from a single family, and as the available clinical, biomarker, and imaging data are limited, the manuscript appears to overstate both the causal relationship between rituximab treatment and the observed clinical course and the mechanistic implications of the findings. The data are more appropriately interpreted as a descriptive and exploratory case series rather than evidence of disease modification by rituximab. Major revision is therefore warranted.	We thank Reviewer 2 for this constructive overall assessment. We agree that the original framing extended beyond what a retrospective observation in two related individuals can support, and we have undertaken a comprehensive revision — detailed point-by-point in R2.1–R2.7 below — that reframes the manuscript throughout as a preliminary, hypothesis-generating familial case series, moderates causal and mechanistic language, and makes the study's limitations explicit at every relevant point (Abstract, Introduction, Results, Discussion, Limitations and Conclusions).

	**Answers, explanations, text amendments**
1. Claims regarding the therapeutic effect of rituximab are overstated. The central concern is that the manuscript overinterprets the therapeutic effect of rituximab in CSF1R-related leukoencephalopathy. Given that this is a retrospective observational report of only two related individuals from a single family, the findings are more appropriately presented as a preliminary familial case series. The overall framing should therefore be moderated. As written, several statements are stronger than the data can support.	**R2.1.** We fully agree and have revised the manuscript title, Abstract, Introduction, Discussion and Conclusions to reframe the report explicitly as a preliminary familial case series and to remove causal language. The revised title (lines 4–6) now reads "Long-term clinical and biomarker observations following rituximab exposure in two siblings with *CSF1R*-related leukoencephalopathy: a preliminary familial case series." The Abstract Conclusions (lines 45–50) now state that observations "cannot be entirely separated from the treatment effect in an uncontrolled observation." Representative examples of moderated language elsewhere include: Introduction (lines 100–103), where a causal statement is replaced by "We hypothesized that depletion of IL-6–producing B-cell subsets may attenuate this inflammatory cycle… Direct evidence for this mechanism in CRL is currently lacking"; Discussion (line 352, opening of the mechanistic paragraph), which now explicitly frames the interpretation as a retrospective integration rather than direct evidence; and the Conclusions (lines 483–489), which now state that the observations "are compatible with, but do not establish, modulation of downstream inflammatory pathways."
2. The familial nature of the case series should be made clear earlier. It should be stated from the outset that the report concerns two siblings from the same family carrying the same heterozygous CSF1R variant. At present, this only becomes fully apparent when the reader reaches the description of Case 2. This point deserves explicit mention in the Abstract and early in the main text, because it affects how the observations are interpreted, including the apparent treatment effect and the extent to which the findings can be generalized.	**R2.2.** We agree and have moved disclosure of the sibling relationship to the earliest possible point in the manuscript. The revised Title (lines 4–6) and Abstract Methods (lines 35–39) now state explicitly that "we describe two siblings from a single family, both carrying the same heterozygous *CSF1R* variant." The Plain Language Summary (lines 52–56) similarly opens with "two sisters with CRL who carried the same disease-causing change." A new closing paragraph has been added to the Introduction making the familial design, shared genetic background and consequent constraints on generalisation explicit before the Methods are reached, and the opening sentence of Results – Case 1 (line 214) now identifies Patient 1 as "the index case… leading to subsequent cascade screening of her sister (Patient 2)" at first mention rather than only when Case 2 is introduced.
3. The treatment rationale and the reason for discontinuing rituximab should be clarified. Because rituximab is not an established treatment for CSF1R-related leukoencephalopathy, the clinical rationale for its use in each case needs to be described more clearly. In particular, it is important to clarify whether rituximab was given under a working diagnosis of multiple sclerosis, or whether it was introduced after recognition of CSF1R-related disease as an off-label, exploratory approach. The reason for stopping treatment after only four infusions should also be stated explicitly. If the authors regard rituximab as beneficial, readers will reasonably ask why it was not continued. Indicating whether discontinuation was planned from the outset or influenced by efficacy, safety, tolerability, or practical considerations would help place the subsequent course in proper context.	**R2.3.** We have clarified both points for each patient. For Patient 1 (lines 243–257), the diagnosis had already been revised to CRL by the time rituximab was initiated in 2015 (two years after genetic confirmation), so rituximab was given as an off-label, exploratory intervention targeting the B-cell–driven inflammatory component of CRL, not under a working MS diagnosis; the regimen was pre-specified by the local multidisciplinary neuroinflammation board as four infusions with planned reassessment, and was not extended because, at reassessment, no new MRI lesions were seen, CSF-CXCL13/CSF-NfL had decreased, clinical status was stable, and the long-term safety of sustained rituximab in a primary microgliopathy is unknown (lines 253–257). For Patient 2 (lines 296–317), the working diagnosis in 2012 was MS, but by rituximab initiation in 2014 the diagnosis had been revised to CRL following cascade screening, so rituximab was likewise introduced under an established CRL diagnosis; discontinuation after four infusions followed the identical pre-specified protocol (lines 314–317). A new Discussion paragraph, "Treatment duration" (lines 371–377), consolidates the rationale for not extending treatment in either patient: pre-specified time-limited design, favourable reassessment, unknown long-term safety of sustained B-cell depletion in a microgliopathy, and the regulatory/reimbursement context for off-label use.
4. The assessment timeline does not allow a meaningful pre-/post-treatment comparison. A key limitation is that the standardized follow-up assessments were performed several years after rituximab exposure rather than before and after treatment. Rituximab was administered in 2015–2017 in Case 1 and 2014–	**R2.4.** We agree entirely. The Methods now state explicitly (lines 128–135) that "we cannot deliver a formal pre-/post-treatment comparison; the data should be interpreted as a descriptive long-term post-exposure trajectory," and that standardised assessments began only from 2020, i.e. three to six years after the final rituximab infusion in each patient. The unavailability of pre-treatment serum NfL (routine clinical use only

	Answers, explanations, text amendments
2016 in Case 2, whereas much of the reported clinical and biomarker follow-up appears to begin only in 2020. As presented, this does not provide a meaningful pre-/post-treatment comparison and therefore substantially limits inference regarding treatment effect. In particular, statements implying that normalization of serum NfL reflects the effect of rituximab should be made more cautiously, since serum NfL was measured only years later. This limitation should be acknowledged explicitly. In addition, the report would be strengthened by clearer presentation of any baseline clinical data available from routine care. Baseline EDSS and cognitive measures such as MMSE would be especially informative for interpreting the later follow-up data.	from 2018) is stated at lines 156–160, and the corresponding caution about serum NfL is repeated at the point of first reporting the value for each patient (Patient 1, lines 264–266; Patient 2, lines 322–324: "serum NfL therefore cannot be used to support inference about an immediate treatment effect"). In direct response to the reviewer's request, we have recovered and reported available routine-care baseline data: EDSS of 4.5 at CRL diagnosis (2013) rising to 6.0 at first rituximab infusion (2015) for Patient 1 (lines 222–226); we note, and now state explicitly, that MMSE was not systematically recorded during routine care in this period. The consolidated Limitations paragraph (lines 447–455) restates these constraints as a single coherent statement of the study's temporal limitations.
5. The interpretation of the clinical assessment results should be more cautious. A clearer explanation of why these particular clinical scales were chosen would be helpful. Although several of the selected measures are understandable in this disease context, the rationale for the full assessment battery is not fully explained. More importantly, the reported scores vary considerably across follow-up, making claims of "stability" or "improvement" difficult to evaluate. For example, HADS scores in Case 1 worsen over time, UPDRS Part III in Case 2 fluctuates substantially, and MMSE changes markedly in both cases. Taken together, these data do not show a consistent pattern of clinical improvement. If the authors wish to retain such claims, they should specify which domains appeared to improve or remain stable, and explain why those changes should be considered clinically meaningful.	**R2.5.** We agree and have added an explicit rationale for the assessment battery to the Methods (lines 133–151), explaining why each instrument (MDS-UPDRS Parts I–IV, modified Hoehn & Yahr, Schwab & England ADL, ALSFRS-R, EDSS, MMSE/SDMT, HADS, MSIS-29/EQ-5D) was selected, while acknowledging that none has been validated specifically for CRL. The Case 1 (lines 273–284) and Case 2 (lines 331–344) narratives have been rewritten to describe a "heterogeneous trajectory rather than a uniform pattern" domain by domain, rather than as a single global claim of stability: EDSS stability is reported separately from the fluctuating MDS-UPDRS Part III scores, the non-directional MMSE/SDMT fluctuations, and the deteriorating HADS scores (cross-referenced to R1.6). The Discussion now contains a dedicated paragraph (lines 378–383) stating explicitly that "we restrict claims of stability or improvement to specific, named domains rather than to the global clinical state," directly addressing the reviewer's request that any retained claims specify which domains improved or remained stable and why this is clinically meaningful.
6. The MRI findings are overinterpreted. The imaging results are interpreted too strongly in support of disease modification. In a progressive leukoencephalopathy such as CSF1R-related leukoencephalopathy, the absence of new focal lesions alone is not sufficient to infer radiological stabilization. Greater emphasis should be placed on atrophy and diffuse white matter degeneration. Notably, the manuscript itself describes enlargement of CSF spaces consistent with atrophy in Case 1, which seems difficult to reconcile with a strong claim of imaging stability. In addition, although REMyDI and MyCPF are interesting exploratory measures, their use as markers of disease progression or treatment response in CRL is not yet sufficiently justified here. A more balanced discussion of what the MRI findings do and do not support would strengthen this section.	**R2.6.** We agree and have substantially rebalanced the radiological interpretation. The MRI Methods (lines 193–202) now state at the outset that the three complementary components of radiological assessment — focal lesion burden, atrophy, and exploratory REMyDI/MyCPF myelin estimation — are not equally validated, and that REMyDI/MyCPF, developed and validated in multiple sclerosis, is exploratory in CRL and should not be used to infer treatment response. The Case 1 imaging narrative (lines 258–262) now explicitly juxtaposes the absence of new focal/enhancing lesions with the documented "widening of cerebrospinal-fluid spaces consistent with progressive cerebral atrophy," and the parallel Case 2 description (lines 325–330) does the same. A new Discussion paragraph, "Imaging biomarkers" (lines 392–403), states directly that absence of new focal/enhancing lesions is "necessary but not sufficient" for radiological stability in a diffuse leukoencephalopathy, restricts the radiological claims to two narrowly defined observations, and notes that MyCPF values (12.2% in 2017 and 13.4% in 2024, Patient 1 only) are reported as methodological proof-of-concept rather than evidence of preserved myelin. This is reiterated in the Limitations (lines 456–461).
7. The proposed mechanism is insufficiently supported by the available data. The Discussion links the apparent effect of rituximab to depletion of IL-6-producing CD20-positive B cells and disruption of an IL-6–STAT3 feedback loop. However, the current data do not directly support this mechanistic interpretation. Neither IL-6 nor STAT3-related markers were measured, and no B-cell phenotyping data are provided. Although NfL and CXCL13 were	**R2.7.** We agree. The Discussion (lines 352–354) now states explicitly that "our mechanistic interpretation is based on a retrospective integration of clinical, radiological, and biomarker data… rather than on direct measurement of IL-6, STAT3 phosphorylation, or B-cell phenotype," and the subsequent mechanistic paragraphs (lines 355–370) use hedged, hypothesis-generating language throughout ("compatible with," "would require measurement… which were not assessed in this study"). We have also made explicit, at line 367 onward, that the asymmetry in biomarker sampling between cases (Case 1 has pre- and post-treatment

	Answers, explanations, text amendments
assessed, these biomarkers are not sufficient to substantiate the proposed pathway. In addition, the biomarker data are not presented in a fully comparable manner across the two cases: Case 1 includes pre- and post-treatment measurements, whereas the corresponding baseline data for Case 2 appear to be unavailable in Table 2, and the values described in Figure 3 seem to have been obtained during treatment rather than before treatment. For this reason, the mechanistic discussion would be more appropriate as a hypothesis than as a conclusion supported by the present report.	CSF values; Case 2 has on-treatment but not pre-treatment values) further limits mechanistic inference. The Limitations section (lines 451–455) now states directly that "mechanistic evidence is lacking as IL-6, STAT3 signaling and B-cell phenotyping were not assessed. The proposed mechanism therefore remains hypothetical and requires validation in future studies."
1. Please clarify which parkinsonism rating scale was used. The manuscript refers to "UPDRS I–VI." If the original UPDRS was used, this should be stated clearly and cited appropriately, with a brief justification for its use. Otherwise, the terminology should be revised to MDS-UPDRS, for which only Parts I–IV apply.	**R2, Minor 1.** We thank the reviewer for identifying this terminological inconsistency. The Methods (lines 139–144) now state that Parkinsonian features were evaluated with the MDS-UPDRS (which comprises only Parts I–IV), and that "the modified Hoehn and Yahr stage and the Schwab and England Activities of Daily Living scale were applied alongside the MDS-UPDRS as freestanding measures,"
2. The reference to Table 2 at line 172 may be incorrect. Table 2 presents CSF biomarker data and does not seem to correspond to the content described in the text. Please check whether a different table was intended.	**R2, Minor 2.** The reviewer is correct; the citation was misplaced. The sentence concerning ACMG/AMP/ClinGen classification of the *CSF1R* variant (Methods, Genetic Analysis) now correctly cites **Table 1** (Demographics and clinical characteristics, which contains the pathogenicity classification), rather than Table 2 (CSF biomarkers before and after rituximab infusion): "The applied criteria and the resulting classification are reported in Table 1" (line 185).
3. The timing of the clinical assessments in Figure 3 should be checked for consistency with the text and Table 3. The legend for Figure 3 indicates that the clinical assessments in Case 2 were performed in 2020, 2021, and 2025, whereas the main text and Table 3 indicate 2020, 2022, and 2025. Please verify and correct these time points as needed.	**R2, Minor 3.** We thank the reviewer for identifying this discrepancy. On re-examination of the source clinical records, we confirm that the correct assessment timepoints for Patient 2 are **2020, 2021 and 2025**, consistent with Figure 3; the earlier references to "2022" for Patient 2 in the main text and Table 3 were transcription errors and have been corrected. Table 1 (line 233, "Time points of clinical evaluations" row) and Table 3 (line 287, "Year" header) now correctly read 2020/2022/2025 for Patient 1 and 2020/2021/2025 for Patient 2, and the Case 2 narrative (line 331) and Abstract/Limitations references to this timepoint have been corrected accordingly.
4. The radar charts may not be the most suitable format for presenting the primary longitudinal data. In a case series of only two patients, a simple longitudinal table may be easier to interpret than a radar chart.	**R2, Minor 4.** We agree that a longitudinal table is the more rigorous format for inspecting individual scale scores in a two-patient series, and note that this is already provided: Table 3 (lines 286–293) presents the full longitudinal numerical data for every clinical instrument and timepoint and, as now stated explicitly in the Methods (lines 152–155), "constitutes the primary quantitative reference for the clinical assessments." We have retained the radar-chart diagrams in Figures 2 and 3 as a secondary, complementary visual representation of the multi-domain profile, rather than substituting them for Table 3, and have clarified this hierarchy (table as primary reference, radar chart as visual summary) in the Methods text.

**Reviewers' comments:**		
**Reviewer #1 (1R), Remarks to the Author**		
	Brief Summary: This manuscript reports the clinical outcomes of two genetically confirmed CSF1R-related leukoencephalopathy (CRL) patients treated with rituximab (RTX), a $CD20^{+}$ B-cell–depleting monoclonal antibody. Both patients, who are siblings carrying the same heterozygous CSF1R c.2562T>A (p.Asn854Lys) variant, received four infusions of RTX at 500 mg. The authors report a heterogeneous clinical trajectory that included stabilization of some clinical measures. The authors propose a mechanistic model in which B-cell–derived IL-6 perpetuates STAT3 dysregulation and white matter injury, and argue that RTX may interrupt this inflammatory cycle.	
	Overall Impression: The authors' revision satisfactorily addresses most concerns that were brought up during the initial review. I recommend acceptance with only a few minor changes.	
1R1	1. Various words do not need capitalization (multiple sclerosis, rituximab, etc.)	We amended the text according to the instructions
1R2	2. Line 37 typo: "L" in "Long-term" should be lowercase	We amended the text according to the instructions
1R3	3. Please ensure all mentions of the gene CSF1R are italicized, while all mentions of the protein CSF1R are not italicized. Most of these are now correct; in line 431, please italicize "CSF1R."	We amended the text according to the instructions
1R4	4. Lines 485-486: "CSF1R-relative neurodegeneration" - if referring specifically to CRL, recommend using the term "CRL" to maintain consistency. Otherwise, please elaborate on difference between CSF1R-related neurodegeneration vs CRL.	We used the term "CRL".
**Reviewer #2, Remarks to the Author**		
	The authors have adequately addressed my major concerns through substantial revisions. In particular, the revised manuscript now clearly acknowledges the limitations of the retrospective familial case-series design, the lack of a formal pre-/post-treatment comparison, the heterogeneous clinical trajectories, the ongoing cerebral atrophy, and the absence of direct evidence for the proposed mechanism. The rationale for rituximab treatment and the decision to limit treatment to four infusions have also been clarified. Overall, the revised manuscript is substantially more balanced and appropriately distinguishes the reported observations from hypothesis-generating interpretations. I have no further comments.	No action taken
